# Rapid hemostasis: a novel and effective outpatient procedure using microwave ablation to control epistaxis of isolated mucosal bulge lesions

**DOI:** 10.1016/j.bjorl.2019.09.002

**Published:** 2019-10-19

**Authors:** Zheng Cai Lou

**Affiliations:** Yiwu Central Hospital, Department of Otorhinolaryngology, Yiwu, China

**Keywords:** Epistaxis, Microwaves, Nasal mucosa, Nasal septal perforation

## Abstract

**Introduction:**

Recurrent epistaxis is commonly encountered in the rhinology outpatient clinic. Under endoscopic guidance, both bipolar cautery and monopolar forceps (combined with suction) have been employed to control the bleeding. However, the use of monopolar forceps requires the placement of grounding pads. Most procedures are currently performed in operating rooms.

**Objective:**

We investigated outcomes after the use of Microwave Ablation (MWA) to control epistaxis in adults with isolated mucosal bulge lesions. All procedures were performed with patients under local anesthesia in our outpatient clinic.

**Methods:**

This is a retrospective cohort study. We included 83 adults with epistaxis of isolated mucosal bulge lesions. Microwave ablation was performed in the outpatient clinic to control bleeding, after induction of local anesthesia. The primary outcome was successful hemostasis. The secondary outcomes were the rebleeding rates at weeks 1 and 4 and month 6, and complications (crust or synechiae formation, septal perforation, and/or orbit or brain complications).

**Results:**

All bleeding points were successfully ablated; hemostasis was achieved within 1–2 min. The mean pain score was 1.83 intra-operatively and 0.95 1 h postoperatively. No patient re-bled, and no severe MWA-related complication (septal perforation, synechiae formation, or orbit or brain complication) was recorded to 6 months of follow-up.

**Conclusions:**

Endoscopic microwave ablation with patients under local anesthesia is a novel, safe, effective, rapid, well-tolerated, outpatient treatment for adults with epistaxis of isolated mucosal bulge lesions, especially those for whom general anesthesia might be risky, those with electrical implants, and those exhibiting contraindications for arterial embolization.

## Introduction

Recurrent epistaxis is commonly encountered in the rhinology outpatient clinic.^1^, ^2^ Either bipolar cautery or monopolar forceps (both accompanied by suction) controls such epistaxis well.^3^, ^4^, ^5^ However, since the latter treatment is compromised by the heat sink effect of local blood flow; it is sometimes difficult to control epistaxis caused by hemorrhage. Also, the technique requires the placement of grounding pads, which carry the risk of burns, interference with pacemaker circuits, and induction of arrhythmia.^6^ Thus, monopolar forceps cannot be used in the outpatient clinic. Although bipolar cautery effectively controls recurrent epistaxis in most cases, neither straight nor curved forceps can readily access the posterior region of a narrow nose, as the bipolar plates are both inflexible and wide.^3^, ^4^ Also, lesion removal and hemostasis cannot proceed using the same instrument; hemostasis must be established after lesion removal.^7^ Thus, bipolar cautery alone sometimes does not control epistaxis if the lesion is large. Radiofrequency Ablation (RFA) has been employed to control hereditary hemorrhagic telangiectasia, but the RFA probe must be maneuvered in combination with an endoscope inside the nasal cavity; this is difficult as the probe is both wide and angled.^7^ Thus, most procedures are performed in the operating room with the patient supine.

Microwave Ablation (MWA) is a special form of dielectric heating, in which a very high but localized temperature rise is induced by absorption of electromagnetic energy at microwave frequencies. The potential benefits include a consistent intratumoral temperature, a large ablation area, rapid ablation, and low periprocedural pain.^8^, ^9^, ^10^ MWA safely and effectively controls active liver hemorrhage, epithelioid hemangioma, and hemorrhaging angiosarcoma.^11^, ^12^, ^13^ MWA does not require the placement of grounding pads. Some studies have suggested that MWA may be useful in cases with contraindications for transarterial embolization, in those unable to undergo angiographic examination, and in those for whom general anesthesia would be risky. MWA can be performed at the bedside and in the emergency department.^12^, ^14^ Recently, MWA has been used to control epistaxis,^15^, ^16^, ^17^ but few publications have reported MWA to control epistaxis in outpatients with isolated mucosal bulge lesions. We evaluated the outcomes of MWA used to control epistaxis in adults with isolated mucosal bulge lesions; MWA was performed in our outpatient clinic with all patients under local anesthesia.

## Methods

The study protocol was reviewed and approved by the Institutional Ethics Review Board of Yiwu Central Hospital (approval nº 20160917). Informed consent was obtained from all participants. The (consecutive) study subjects were adults with epistaxis of isolated mucosal bulge lesions who visited the Department of Otorhinolaryngology and Head-and-Neck Surgery of Yiwu Central Hospital between January 2017 and December 2017. The inclusion criteria were: (1) Idiopathic adult epistaxis with or without anterior/posterior nasal packing, (2) stable vital signs, (3) endoscopic identification of a bleeding point on an isolated mucosal bulge lesion (a primary telangiectasia, or an isolated mucosal bulge with a red or white top). The exclusion criteria were traumatic epistaxis, a bleeding tumor, postoperative epistaxis, or bleeding diathesis. All patients underwent preoperative laboratory testing to exclude any bleeding or coagulation disorder.

### Technical details

**Figure 1:**
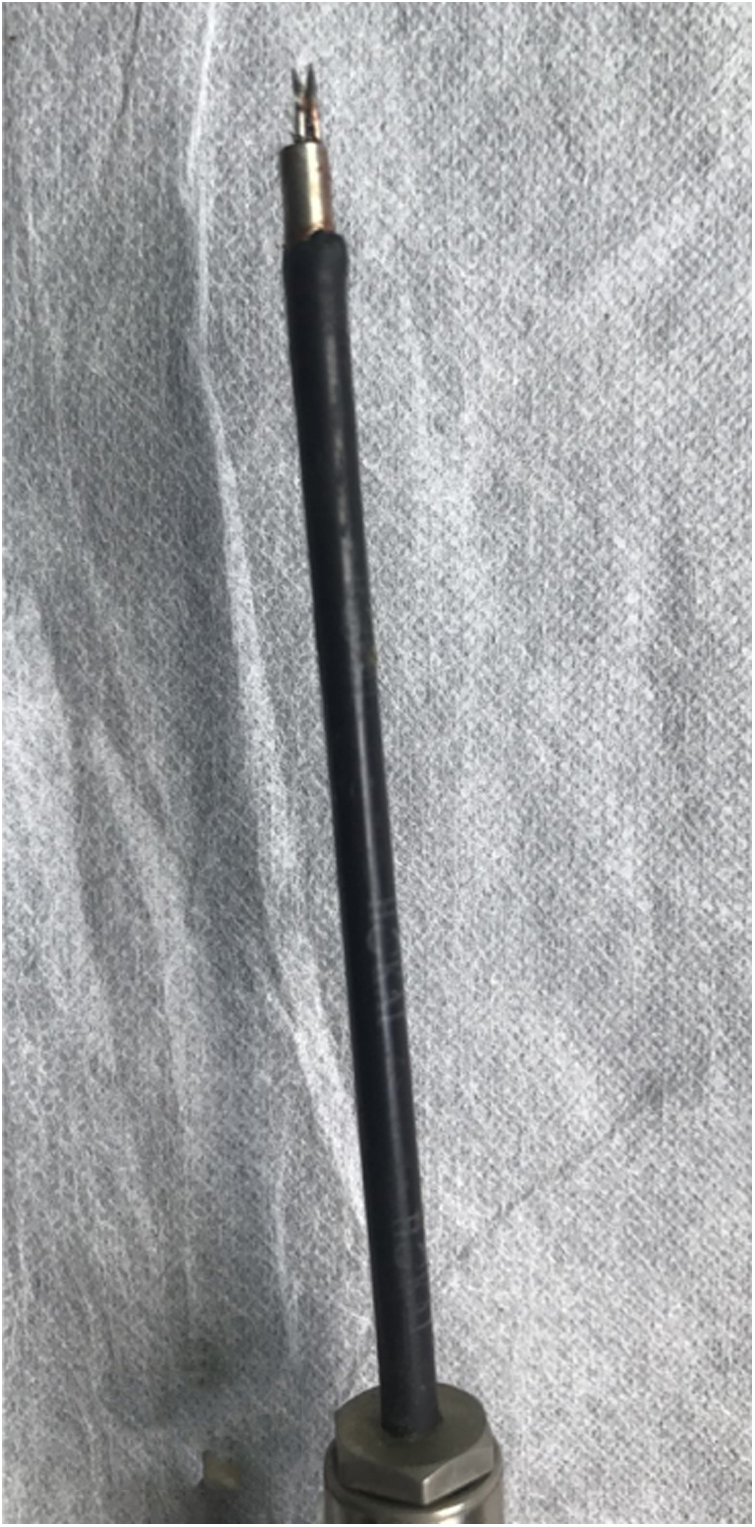
Detailed picture of the microwave antenna tip.

**Figure 2:**
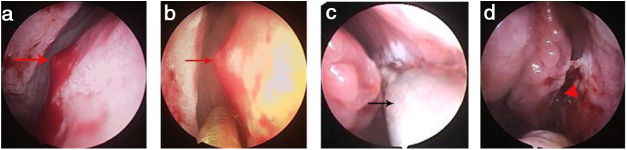
Bleeding point on the upper nasal septum. (a) Bleeding point (actively bleeding); (b) Bleeding point (bleeding ceased); (c) MWA; (d) The MWA zone. Red arrows, bleeding point; red triangle, ablation zone; black arrows, microwave antenna.

All patients were placed in the sitting position. Bleeding sites/points were identified endoscopically with patients under local anesthesia. Pre-existing nasal packing and blood clots were removed. Cotton pledgets soaked in 4% (w/v) lignocaine with adrenaline were placed in the nasal cavity to improve visibility and reduce bleeding. Possible bleeding sites were examined in the following order using an endoscope and suction: the anterior nasal septum, olfactory cleft, middle meatus, inferior meatus, anterior roof of the nasal cavity, bottom of the common nasal meatus and nasopharynx. MWA was performed using an EBH-IV Microwave Therapy instrument (Zhuhai Hokai Medical Instruments Co., Ltd., China) featuring a 2450 MHz cooled-shaft antenna to control bleeding after the identification of bleeding points on isolated mucosal bulge lesions. The MWA output power was 60–80 W. The microwave antenna used was 9 cm in length and 3 mm in outer diameter, similar in dimensions to the bipolar cautery or monopolar suction instrument (Fig. 1); the antenna readily attained the anteroinferior part of the nasal septum (under the endoscope) (Fig. 2). The antenna tip used was a split-type double needle that points upward. The needle width was 1 mm and the length of the upward-pointing tip was 2 mm. That part of the tip was used to contact the nasal mucosa or the bulge lesion. A footplate-operated switch was used to control ablation time and the length, width, and depth of the thermal lesion. The device immediately ceases ablation if the footplate is deactivated. The MWA time was 1–3 s; repeat ablation of the same lesion was allowed.

In patients with inactive bleeding, each bleeding point was ablated under endoscopic guidance in a distal-to-proximal manner to create a uniform grey/white-colored ablation zone covering both the lesion and the periphery. If a lesion bled excessively during MWA, or was actively bleeding, the MWA antenna was placed directly into the lesion and then rapidly removed to reduce blood flow.^15^ Cotton pledgets soaked in 4% (w/v) lignocaine with adrenaline were placed in the ablation zone for a few minutes, the bleeding point and ablation zone were further observed endoscopically, and those regions were then re-ablated until a grey/white-colored ablation zone formed (Fig. 2 and Supplementary Material – Video).

All patients were discharged on the day of MWA. Outpatient followup was scheduled at 2 and 4 weeks and 6 months. The nasal cavity was re-examined at each followup. The primary outcome was successful hemostasis. The secondary outcomes were the rebleeding rates at 2 and 4 weeks and 6 months, and complications (crust or synechiae formation, septal perforation, and orbit or brain complications). All patients were asked to score pain on a Visual Analog Scale (VAS) ranging from 0 to 10 both intra-operatively and 1 h postoperatively.

## Results

**Figure 3:**
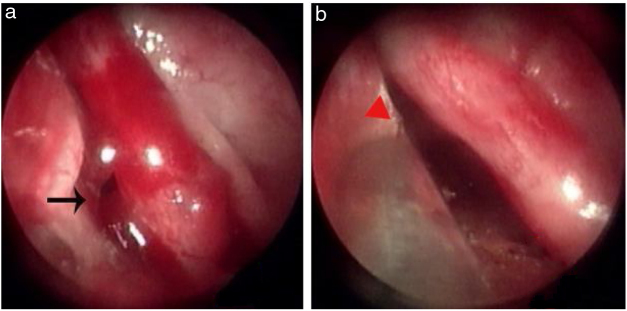
Bleeding point on the upper nasal septum. (a) Bleeding point; (b) The MWA zone. Black arrows, bleeding point; red triangle, ablation zone.

**Figure 4:**
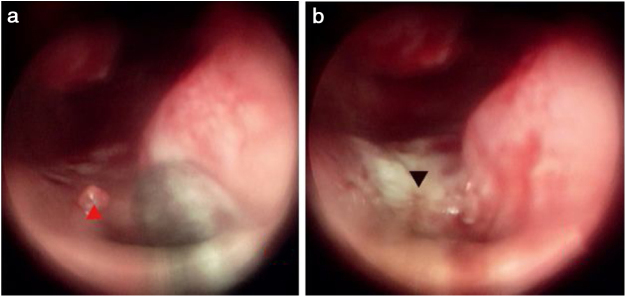
Bleeding point on the bottom of the common nasal meatus. (a) Bleeding point; (b) The MWA zone. Red triangle, bleeding point; black triangle, ablation zone.

A total of 83 patients with idiopathic epistaxis met the inclusion criteria. All bleeding points were on unilateral isolated mucosal bulge lesions. Fifty-two patients were male and 31 female; 49 had left-side lesions and 34 had right-side lesions. The average age was 42.4 ± 5.1 years (range, 28–75 years); the duration of epistaxis was 4.7 ± 2.8 days (range, 2–11 days). Of the 83 patients, 32 had previously undergone anterior nasal packing, and 19 had undergone both anterior and posterior nasal packing. The bleeding site/point was on the upper nasal septum in 47 (56.6%) (Figure 2, Figure 3), the bottom of the common nasal meatus in two patients (2.7%) (Fig. 4), the inferior meatus in 11 (14.7%), the mucosal groove of the middle meatus in 8 (10.7%), the concave side of the medial region of a deviated nasal septum in 12 (16.0%), and the anterior roof of the nasal cavity in 3 (4.0%). All bleeding points were successfully ablated by MWA; hemostasis was achieved within 1–2 min. All patients were cooperative. The mean pain score was 1.83 intra-operatively and 0.95 1 h postoperatively. At the 2 week follow-up, only 18% of patients exhibited some crusting; at the 4 week follow-up, all nasal mucosae exhibited normal morphology. No patient experienced recurrent epistaxis within 6 months. No severe MWA-related complication (septal perforation, synechiae formation, or orbit or brain complication) was noted to the 6 month follow-up.

## Discussion

To achieve hemostasis of idiopathic recurrent epistaxis, it is essential to endoscopically identify the bleeding sites/points. Anterior and posterior packing and arterial ligation or embolization of the responsible vessel were previously used to treat recurrent epistaxis when the bleeding sites were unknown.^18^, ^19^ However, electrocoagulation (bipolar cautery or monopolar forceps, both with suction) is now commonly used to treat epistaxis when the bleeding sites are known.^3^, ^4^, ^5^, ^6^ All of these procedures are usually performed in the operating room, for both subjective and objective reasons. MWA was initially developed in the early 1980s to allow hemostasis along the plane of transection during hepatic resection,^20^ and is excellent for that purpose.^10^, ^11^, ^12^, ^13^ The MWA antenna greatly raises the temperature of the target tissue within seconds, creating a large ablation area within a short time.^8^, ^9^, ^10^ Because MWA is minimally affected by the extent of tissue perfusion, it is especially useful when treating organs exhibiting high-level perfusion or ones that adjoin vascular heat sinks.^21^ MWA has been shown to form large ablation areas within targeted zones adjacent to vessels (thus in the presence of blood perfusion); thus, MWA is ideal for the treatment of vascularized tissue.^22^ Both RFA and electrocautery are compromised by blood perfusion and blood flow.^21^ Both our present study and others have found that most bleeding points are associated with isolated mucosal bulge lesions with red or white tops, including primary telangiectasias.^5^, ^23^ Histologically, most bleeding points exhibit telangiectasic features and capillary hemangiomas.^23^, ^24^ Thus, MWA is theoretically better than electrocautery for the treatment of epistaxis associated with large vascular lesions. We successfully treated all 83 patients within 1–2 min. Each thermal lesion was approximately 2 mm in length and 1 mm in width, identical to or slightly exceeding the length and width of the upward-pointing portion of the microwave antenna tip. The depth of thermal penetration was approximately 0.5–1 mm.^16^ MWA is very unlikely to injure the orbit or brain, or to create a septal perforation. No severe MWA-related complication (septal perforation or orbit or brain complication) was recorded to the 6-month follow-up.

As a novel form of epistaxis therapy, MWA offers real advantages over conventional electrocautery: (1) Electrocautery raises the temperature to over 400 °C, usually associated with skin burns and local pain.^7^ However, MWA raises the temperature to only 60–100℃, causing less procedural pain.^9^ All patients cooperated well; the mean intra-operative pain score was 1.83. Thus, the use of MWA to treat epistaxis is appropriate for patients for whom general anesthesia is risky. (2) Bipolar cautery creates a thermal injury featuring secondary black crusting^7^: the hot probe tends to adhere to the tissue coagulum, triggering re-bleeding when the probe is removed.^6^ However, MWA is not associated with secondary crusting. Nevertheless, a few yellow crusts were evident in 18% of our patients. It is possible that long-duration ablation of the same area raised the temperature to over 100 ℃, creating a coagulative region featuring tissue charring.^25^ Thus, repeat brief ablations are preferred. However, none of our 83 patients reported recurrent epistaxis during 6 months of follow-up. (3) MWA is less expensive than bipolar cautery (approximately US$11.40 vs. $19.10, respectively, in our department), because the MWA antenna is re-usable but the bipolar cautery device is not. (4) The external diameter of the MWA antenna is similar to that of monopolar forceps, and thus accesses all areas of the nasal cavity, overcoming the limits imposed by the bipolar plates in narrower nasal cavities. (5) Bipolar cautery (lesion removal) must be followed by hemostasis.^7^ In contrast, MWA both removes the lesion and establishes hemostasis.^2^ (6) Electrocautery requires the placement of grounding pads, the patient must be supine and the procedure may interfere with pacemaker circuits and induce arrhythmia.^6^, ^14^ Electrosurgery cannot be performed in outpatient clinics or on patients with electrical implants. The temperature attained during MWA is lower than that attained during electrocautery, and grounding is not required. Finally, MWA can be delivered in the outpatient clinic, even to patients with implants such as pacemakers.^12^, ^14^

## Conclusion

Microwave ablation is a suitable method to treat epistaxis in selected patients with minimal pain and short ablation time. It can be used in the outpatient setting to control adult epistaxis of isolated mucosal bulge lesions, especially in patients for whom general anesthesia might be risky, those with electrical implants and those contraindicated for arterial embolization.

## Conflicts of interest

The authors declare no conflicts of interest.
